# Validity and Reliability of the Korean Version of the Weight Self-Stigma Questionnaire (WSSQ-K)

**DOI:** 10.3390/nursrep13020073

**Published:** 2023-05-26

**Authors:** Seyoen Park, Kawoun Seo

**Affiliations:** 1 College of Nursing, Chungnam National University, Daejeon 35015, Republic of Korea; 2 Department of Nursing, Joongbu University, Chungnam 32713, Republic of Korea

**Keywords:** body weight, self-stigma, questionnaires, validity, reliability

## Abstract

Weight self-stigma refers to negative self-prejudice due to the internalization of negative social messages about one’s weight. People with high self-stigma may have low self-esteem and decreased social activity. Weight-related self-stigma can create diet-related disorders since it is highly related to the recognition of body types. However, there are no tools available to measure the weight-related stigma of the general public in Korea. This study evaluated the validity and reliability of the Korean version of the Weight Self-Stigma Questionnaire (WSSQ-K). A methodological study was conducted with 150 Korean university students. Construct validity was evaluated using exploratory factor analysis. The WSSQ-K was correlated with body mass index and measures of self-esteem and weight concern to evaluate concurrent validity. Internal consistency reliability was evaluated with Cronbach’s alpha. In the exploratory factor analysis, two factors were proposed: “self-devaluation” (Cronbach’s α = 79) and “fear of enacted stigma” (Cronbach’s α = 82). Factor loadings for the 12 items on two factors ranged from 0.539 to 0.811, which explained 53.3% of the total variance. The WSSQ-K correlated with body mass index, self-esteem, and weight concern. The findings showed that the WSSQ-K was a reliable and valid measure that could be used for evaluating weight self-stigma in normal-weight adults in Korea.

## 1. Introduction

Weight self-stigma refers to weight-related negative self-bias due to the internalization of negative social messages about one’s weight [1]. This was mainly established when Global North regions, such as North America, Australasia, and Western Europe, internalized negative images, such as laziness and lack of self-control, as symbols for an obese body [2]. This weight-related self-stigma manifests itself in individuals as discrimination, neglect, and bullying [2] and lowers their quality of life. It has also become a major public health issue because it not only affects health-related behaviors but also has negative lifetime impacts [3].

However, unlike the Global North, where the weight-related stigma on obesity is a problem, in Korea, weight-related stigma is a problem that has spread throughout society. The reason is that interest in health, weight, and appearance has been very high in Korea recently [4]. Contrary to the World Health Organization (WHO) standard, which defines a body mass index (BMI) of 25 kg/m^2^ or more as overweight and 30 kg/m^2^ or more as obese, Korea classifies 23 kg/m^2^ or more as overweight and 25 kg/m^2^ or more as obese. This is because, unlike in the West, many reports indicate that the risk of complications of metabolic syndrome, such as hypertension and diabetes, already increases in Asians from a body mass index of 25 or less [5]. Therefore, Korea does not apply the WHO’s standards but applies the Asia-Pacific standards established in 2000 [6]. The prevalence of obesity in Korea is only 4.7%, based on a BMI of 30 kg/m^2^ or higher, which meets the WHO and Korean obesity standards [7]. However, the weight control rate is very high compared to the world [7], indicating that Korean society is sensitive to body shape.

Individuals with a high BMI have a higher risk of cardiovascular diseases and lifestyle disorders than those with a normal weight [8]. Similarly, underweight individuals have an increased risk of cardiovascular disease and death because their bones, muscles, and organs become weak [9]. Therefore, maintaining a normal BMI range is essential for a healthy life. However, even normal-weight individuals may perceive themselves as underweight or overweight if their subjective perception of body shape is distorted [10]. Low satisfaction with appearance can lead to lower self-esteem, increased depressive symptoms, and a lower quality of life [5,11,12]. In addition, people with low body shape satisfaction are at risk of becoming underweight or overweight due to poor weight management and, in severe cases, eating disorders [13]. Given the study of eating problems and health-related problems caused by overeating in Korea [14,15], it is necessary to reliably evaluate individuals’ beliefs about weight so that they can be understood and addressed when justified.

Among the tools developed to evaluate weight bias, the Weight Concern Scale [4] and Weight Bias Measurement Tool [16] were translated into Korean and showed excellent validity and reliability. However, as these tools focus on social stigma related to obesity or weight, they have limitations in measuring other dimensions of weight self-stigma, such as shame or negative self-assessment [1]. The Weight Self-Stigma Questionnaire (WSSQ) was developed by Lillis et al. [1]. It measures weight-related self-stigma using 12 items. WSSQ has been translated and used in various countries including Germany, Turkey, Hong Kong, China, and France. In addition to its validation on overweight children and adolescents, this instrument underwent validation on a sample of children and adolescents with normal weight in Hong Kong [17]. The study involved administering the WSSQ to 287 individuals in this group, aged between 8 and 12. Confirmatory factor analysis was conducted after dividing the participants into overweight and normal weight groups. The results showed that the structural validity of the instrument was confirmed across both weight-related groups [17].

Considering the issues related to Koreans’ weight, self-stigma measures should be evaluated and managed for the psychological well-being and health of people with weight-related notions. However, despite the WSSQ showing good validity and reliability in measuring body weight self-stigma, it has neither been translated into Korean nor validated for use among Korea’s general population. Therefore, this study tried to evaluate whether the Korean version of the WSSQ (WSSQ-K) was suitable for measuring the degree of self-stigma related to weight by verifying its reliability and validity.

## 2. Materials and Methods

### 2.1. Design

This study used a methodological approach to translate the WSSQ into Korean and evaluate its psychometric properties.

### 2.2. Participants and Data Collection

The study participants were university students attending universities located in C and D cities. Data were collected from 1 November to 10 December 2019. Only those who voluntarily agreed to participate in the study after receiving information about the necessity and purpose of the study were included in the study. Those who could not communicate or had cognitive decline were excluded from the study. The 12-item WSSQ required 120 participants since exploratory factor analysis requires a sample size that is at least 5–10 times the number of items on the scale [18]. However, after anticipating potential problems with response fidelity, the questionnaire was distributed to 150 students, and their survey results were used for analysis.

### 2.3. Ethics Considerations

This study was conducted after obtaining approval for the research proposal from the Institutional Review Board of J University (JIRB-2019090301-02-190930). Before data collection, a professor at the university explained the study’s purpose, necessity, and content to students, and their consent was obtained. The consent form enumerated the survey’s background and purpose and clauses regarding anonymity and confidentiality. It also stated that the survey results would not be used for purposes other than the study, questionnaires will be destroyed at the end of the study, and participants could withdraw their consent at any time. Participants received mobile coupons for coffee as an incentive to participate. The completed questionnaires were collected separately from the consent forms and were stored in a locked cabinet until it was time for the results to be anonymously electronically coded and analyzed.

### 2.4. Measurements

#### 2.4.1. Weight Self-Stigma

The Korean version of the WSSQ [1] was prepared through translation and reverse translation with the original developer’s permission. The tool was modified to be applied and used in the Korean context through content validity. The original WSSQ consisted of 12 items: six items on self-worth reduction and six items on fear of stigma recognition. Responses to the items were provided on a 5-point Likert scale. Higher total scores indicated higher weight self-stigma. The original tool’s Cronbach’s α was 0.88 [1].

#### 2.4.2. Self-Esteem

Self-esteem was measured using the Rosenberg Self-Esteem Scale (RSES), developed by Rosenberg [19] and adapted by Lee et al. [20] This tool contained 10 items: six items on positive self-esteem and four on negative self-esteem. Items were responded to using a 4-point Likert scale. Negative self-esteem items were reverse scored. Higher scale scores indicated higher levels of self-esteem. In Lee et al.’s [20] study, Cronbach’s α was 0.81, while α was 0.80 in the present study.

#### 2.4.3. Weight Concern

The Korean version of the Weight Concern Scale, developed by Killen et al. [21] and translated into Korean by Seo et al. [4], was used in this study. The scale was developed to screen for students at risk for an eating disorder. There were five questions about the participant’s weight and body type, fear of gaining 1 kg of weight, weight importance, and feeling obese when finally dieting. The higher the score the higher the level of weight-related concern. The internal consistency coefficient was reported as 0.85 [21], while a domestic validity study by Seo et al. [4] reported a coefficient of 0.73. In this study, Cronbach’s α was 0.75.

### 2.5. Procedure

#### 2.5.1. Translation Process

The WSSQ was translated using the reverse translation process and modified to suit the target participants. First, two individuals—a nursing professor who was fluent in both Korean and English and a nursing doctoral student in Korea—independently translated the items from the WSSQ into Korean. Next, two translators compared and reviewed the translated versions, and items were revised through discussion. The tool was then translated back into English by a native English speaker fluent in Korean and unfamiliar with the original version. The reverse-translated tool was compared with the original by a native English speaker to verify whether there were any differences in the items’ meanings.

#### 2.5.2. Content Validity

To evaluate content validity, the translated tool was shown to three nursing professionals. They compared the original with the translated tool to determine whether the meaning of each of the items was retained, the words were appropriate, and any questions would be difficult for the target participants. We asked those who reviewed the items to revise them so that they could be extended and applied. Consequently, some items were revised based on their feedback. For example, the statement “I became overweight because I am a weak person” was modified to “The reason I am at my current weight is because my will is weak”.

#### 2.5.3. Face Validity

Before conducting the survey, face validity was determined by administering the measure to 10 university students who were not included in the final sample. The students evaluated the readability, understanding, and appropriateness of the WSSQ-K items. Questionnaire preparation time and reactions while completing the questionnaire were observed; if an item was unclear, the participant was asked to provide an opinion. No changes were made in the process, and the questionnaire took about 3–5 min to complete.

#### 2.5.4. Item Analysis

Item analysis involved determining the mean, standard deviation, skewness, and kurtosis of each item and examining the degree of partiality of each item. The item–total correlation coefficient and the alpha of the item deleted were checked. Items with a correlation coefficient of less than 0.30 and items that increased the reliability coefficient upon deletion were discarded [22].

#### 2.5.5. Construct Validity

Exploratory factor analysis (EFA) was used to investigate the construct validity of the WSSQ-K. Principal component analysis was used to verify the underlying structure, while the varimax orthogonal rotation method was used to minimize the number of factors and maximize factor loadings of the items on each factor. The total variance was based on 50% or more, and factors with an initial eigenvalue of 1.0 or more were extracted. The commonality and factor loadings were checked, and items with factor loadings under 0.30 were removed [23].

#### 2.5.6. Concurrent Validity

Concurrent validity was verified by correlating the WSSQ-K with BMI, Self-Esteem, and Weight Concern Scale. Weight self-stigma has been found to be related to BMI, self-esteem, and weight concern in a previous study [24]. A BMI of 23 kg/m^2^ or more was classified as overweight, and a BMI of 25 kg/m^2^ or more was classified as obese according to the Asia-Pacific perspective [6]. Self-esteem was measured using the 10-item Rosenberg Self-Esteem Scale, and weight concern was measured using the Korean version of the 5-item Weight Concern Scale.

#### 2.5.7. Reliability

Internal consistency reliability of the WSSQ-K was evaluated by calculating Cronbach’s alpha.

### 2.6. Data Analysis

The collected data were analyzed using the SPSS/WIN 24.0 program. Descriptive statistics were used for the general characteristics of the participants, and the mean and standard deviation for each item of the WSSQ-K, skewness and kurtosis, item–total correlation coefficients, and changes in reliability with item deletion were analyzed. To verify the suitability of the EFA, Keiser–Meyer–Olkin (KMO) and Bartlett sphericity tests were performed. Principal component analysis with varimax rotation was used to evaluate construct validity. Concurrent validity was evaluated by correlating the WSSQ-K with the measures of BMI, self-esteem, and weight concerns using Pearson’s correlation coefficient, and it was confirmed that the correlation coefficient was statistically significant with a value of 0.03 or higher. Cronbach’s alpha was calculated to evaluate the internal consistency reliability of the WSSQ-K.

## 3. Results

### 3.1. Participants’ Characteristics

Among the participants, 72.0% were women, and 28.0% were men; the average age was 21.6 ± 1.95 years. The number of participants with normal weight was 44.0%, while 34.7% were underweight, and 21.3% were overweight. Only 12.7% (19) participants were satisfied with their weight, while 40.0% were satisfied with their overall lives. Additionally, 60.7% of participants indicated they had a positive character, 75.3% reported that their subjective economic status was moderate, and 60.7% answered that they had good interpersonal relationships.

### 3.2. Item Analysis

The results of the item analysis indicated that the average item score was 1.60–3.86, and the standard deviation ranged from 0.73 to 1.19. If the absolute value of skewness was 2 or less and the absolute value of kurtosis was 7 or less, the normal distribution was satisfied (24). In this study, the absolute value of skewness was 0.07 to 1.56, and the absolute value of kurtosis ranged from 0.50 to 2.91. Most correlation coefficients between the items and the total score ranged from 0.35 to 0.68, and none were less than the standard value of 0.30. Regarding item deletion, there were no items that increased the reliability when deleted. Thus, all 12 items were used for the exploratory factor analysis (Table 1).

### 3.3. Construct Validity

Before exploratory factor analysis, KMO and Bartlett’s sphericity tests were conducted to confirm whether the data were suitable for factor analysis. The KMO value was 0.801, indicating that a common potential factor existed, and Bartlett’s sphericity test had a significance level of *p* < 0.001, which indicated that factor analysis of the data may have been useful.

To verify the construct validity of the 12 items, principal component analysis was used as the factor extraction method, and an exploratory factor analysis was performed using varimax as the rotation method. Initially, the principal component analysis was performed without rotation. Therefore, it was appropriate to divide the 12 items into two or three factors based on an eigenvalue of 1.0 or more and a cumulative explanatory power of 50% or more. After subdividing them into two factors, the cumulative explanatory power became 53.3%. Afterward, varimax rotation was performed for the convenience of interpretation, and the commonalities of the 12 items were 0.381 to 0.696. Factor loadings on factor one (six items) ranged from 0.558 to 0.811, the eigenvalue was 3.19, and the explanatory power was 26.55%. The items belonging to this factor showed that feelings of guilt or remorse were the cause of the participants’ weight problems and comprised items on the devaluations of one’s will and ability. Therefore, factor one was named “self-devaluation”, as in previous studies. The factor loadings on factor two (six items) ranged from 0.539 to 0.772, the eigenvalue was 3.21, and the explanatory power was 26.74%. The items were about anxiety due to weight problems and being discriminated against or being worried about social/relationship problems. Therefore, factor two was named “fear of enacted stigma”, as in the original WSSQ (Table 2). The two factors formed the subscales of the WSSQ-K.

### 3.4. Concurrent Validity

BMI positively correlated with the WSSQ-K total scale scores (r = 0.36, *p* < 0.001), self-devaluation subscale scores (r = 0.66, *p* < 0.001), and fear of enacted stigma subscale scores (r = 0.23, *p* = 0.005). Conversely, self-esteem negatively correlated with WSSQ-K total scale scores (r = −0.25, *p* < 0.001), self-devaluation scores (r = −0.18, *p* = 0.029), and the fear of enacted stigma scores (r = −0.28, *p* = 0.001). There were significant positive correlations between weight concern and WSSQ-K total scale scores (r = 0.52, *p* < 0.001), self-devaluation scores (r = 0.53, *p* < 0.001), and fear of enacted stigma scores (r = 0.37, *p* < 0.001) (Table 3).

### 3.5. Reliability

Cronbach’s α was 0.85 for the WSSQ-K total scale. The self-devaluation subscale α was 0.79, and the fear of enacted stigma subscale α was 0.82 (Table 2).

## 4. Discussion

This study was conducted to evaluate the validity and reliability of the Korean version of the weight self-stigma questionnaire (WSSQ-K) developed for underweight or overweight people by applying it to normal weight adults. In this study, as a result of classifying the sub-factors of the WSSQ-K using EFA, they were divided into two sub-domains, self-depreciation factors and fear factors of enacted stigma, similar to the existing tools [1]. Thus, the results of the EFA in the present study were consistent with the findings of the original tool, as well as with the versions from Germany [25], Turkey [26], and China [27]. The original tool was designed to measure multidimensional characteristics of weight self-stigma, targeting overweight or obese people [1]. However, despite expanding the target population to include normal-weight individuals, the tool’s validity remained the same in this study. Consequently, the WSSQ-K demonstrated utility as a measure of weight self-stigma for individuals with normal weight. Considering that Koreans generally have a high rate of weight loss attempts despite being a low-BMI population [28], it can be assumed that the ideal weight for Koreans is underweight. In a previous qualitative study, the findings showed that Koreans were envious of Korean celebrities’ bodies and were overly concerned with receiving weight-related attention [29]. Given the social context, this may explain why weight self-stigma appears more often in the Korean general population than in the West, where weight self-stigma occurs mainly in overweight or obese individuals [12].

The results of the concurrent validity analysis showed that the WSSQ-K was significantly positively correlated with BMI and weight concerns, which was consistent with the studies by Lillis et al. [1] and Sevincer et al. [26]. However, there was no significant relationship between the WSSQ and BMI in a sample of overweight/obese French adolescents [26]. The different findings in the correlates of weight self-stigma could be due to cultural differences in the populations studied [26] or due to a variety of other characteristics. However, little research has been conducted on weight self-stigma to date. Thus, a follow-up study on continuous weight self-stigma is needed. In the present study, participants had a relatively lower BMI than in the other studies; however, a higher BMI was still associated with higher weight self-stigma. This suggests Koreans may be relatively more sensitive to weight stigma compared with people from the West, regardless of whether they are underweight, normal weight, or overweight. Additionally, participants with high-weight self-stigma showed low self-esteem and high weight concern, which was consistent with Lillis et al.’s findings [5]. Previous research showed individuals with high self-stigma had low self-esteem, and people with high concern about their weight and body shape were more likely to have weight control behaviors due to low body satisfaction [4]. Considering that measuring weight concern was designed to screen students at risk of developing an eating disorder, the weight self-stigma tool measured weight change, feelings about weight, and perceived judgment from others. Therefore, this tool is useful for measuring an individual’s weight bias among people with a range of BMI levels.

The internal consistency reliability of the WSSQ-K was good. Cronbach’s α coefficients were 0.85 for the total scale, 0.79 for the Self-Devaluation subscale, and 0.81 for the Fear of Enacted Stigma subscale. For the original tool, Cronbach’s α was 0.87 for the total scale, while the scales measuring self-devaluation and fear of enacted stigma were 0.81 and 0.87, respectively [1]. Additionally, in the German version of the WSSQ, Cronbach’s α was 0.87 for the total scale, and the scales measuring self-devaluation and fear of stigma were 0.74 and 0.83, respectively [25]. Therefore, the internal consistency reliability of the WSSQ-K was “acceptable” according to Nunnally and Bernstein [22] because the Cronbach’s α coefficients were higher than 0.70.

The study had limitations. First, the study participants were limited to university students in two cities. Future research should include various regions and age groups in Korea. Second, the test–retest reliability of the WSSQ-K was not examined and should be assessed to evaluate the tool’s stability. Nevertheless, the reliability and validity of the tool were verified as a measure of weight self-stigma. Therefore, the WSSQ-K had utility for assessing the level of self-stigma with weight-related issues in underweight, normal weight, overweight, and obese men and women in Korea. Using this tool, clinicians could objectively evaluate the weight-related self-stigma of Korean-speaking individuals in the future. The tool could also help healthcare providers in understanding individuals with weight-related self-stigma. It could also be used to create educational materials on weight-related self-stigma not only for medical personnel but also for pre-medical personnel and the general public. In terms of research, it is possible to prepare basic data for interventions to reduce weight-related self-stigma by exploring its affecting factors.

## 5. Conclusions

This study translated the WSSQ and verified the validity and reliability of the WSSQ-K. The original tool consisted of two factors and 12 items. Results of the factor analysis indicated that the factor structure of the WSSQ-K was consistent with the original version. Additionally, the WSSQ-K’s suitability was verified by demonstrating content validity, face validity, construct validity, reliability, and concurrent validity. Therefore, the WSSQ-K should contribute to the development and evaluation of programs to correct weight-related biases or discrimination by allowing for the assessment of weight-related self-stigma in the Korean student population.

## Figures and Tables

**Table 1:** Results of the item analysis of the WSSQ-K (*N* = 150).

No.	M ± SD	Skewness	Kurtosis	Corrected Item Total Correlation	α If Item Deleted
1	2.96 ± 0.88	0.19	−0.58	0.44	0.84
2	3.86 ± 0.73	−0.99	2.46	0.39	0.84
3	2.78 ± 1.13	0.09	−0.97	0.64	0.82
4	3.08 ± 1.19	−0.14	−1.00	0.55	0.83
5	3.28 ± 1.10	−0.13	−0.89	0.48	0.83
6	2.83 ± 1.10	0.12	−0.94	0.50	0.83
7	2.84 ± 0.98	0.07	−0.64	0.54	0.83
8	2.02 ± 1.00	1.00	0.82	0.56	0.83
9	1.60 ± 0.79	1.56	2.91	0.35	0.84
10	2.40 ± 1.12	0.52	−0.50	0.68	0.82
11	2.86 ± 1.18	−0.12	−1.07	0.52	0.83
12	1.77 ± 0.82	0.94	0.76	0.48	0.83
Total scale	2.67 ± 0.57	0.00	0.01		

**Table 2:** Descriptive statistics and results of exploratory factor analysis of the WSSQ-K (*N* = 150).

No.	Communalities	Self-Devaluation	Fear of Enacted Stigma
4	0.668	0.811	0.101
2	0.598	0.766	−0.107
3	0.619	0.731	0.290
1	0.415	0.633	0.122
5	0.381	0.564	0.251
6	0.396	0.558	0.292
10	0.696	0.315	0.772
12	0.582	0.067	0.760
9	0.583	−0.113	0.755
8	0.598	0.189	0.750
11	0.437	0.307	0.585
7	0.422	0.363	0.539
Eigenvalue		3.19	3.21
Variance, %		26.55	26.74
Cumulative variance, %		26.55	53.30
M ± SD		3.13 ± 0.72	2.25 ± 0.72
Cronbach’s α (total = 0.85)		0.79	0.82

The extraction method was by principal component analysis with varimax rotation. KMO = 0.801, Bartlett’s test < 0.001. WSSQ-K, Korean version of the Weight Self-Stigma Questionnaire, M ± SD, mean ± standard deviation.

**Table 3:** Correlations with the WSSQ-K (*N* = 150).

	WSSQ-K (12 Items)		
Variable	Total Scale	Self-Devaluation Subscale	Fear of Enacted Stigma Subscale
Body mass index	0.36 (<0.001)	0.66 (<0.001)	0.23 (0.005)
Self-esteem	−0.25 (<0.001)	−0.18 (0.029)	−0.28 (0.001)
Weight concern	0.52 (<0.001)	0.53 (<0.001)	0.37 (<0.001)

WSSQ-K, Korean version of the Weight Self-Stigma Questionnaire. *p*-values are in parentheses.

## Data Availability

The data that support the findings of this study are available upon request to the corresponding authors.
